# Role of Entropy–Enthalpy Competition on the Thermochemically Driven Shape Memory Effect in Amorphous Polymer Films

**DOI:** 10.3390/ma18071630

**Published:** 2025-04-03

**Authors:** Jianing Shi, Yong Liu, Xinhui Peng, Zhe Li, Xiaodong Wang

**Affiliations:** 1 School of Materials Science and Engineering, Zhejiang University, Hangzhou 310027, China; shijianing.com@163.com (J.S.); liuyong.mse@zju.edu.cn (Y.L.); 2 Hebei Key Laboratory of Mechanical Reliability for Heavy Equipments and Large Structures, School of Civil Engineering and Mechanics, Yanshan University, Qinhuangdao 066004, China; pengxinhui@stumail.ysu.edu.cn (X.P.); lz0503@ysu.edu.cn (Z.L.)

**Keywords:** shape memory polymer, thermodynamics, thermochemical field, shape memory effect

## Abstract

The application of thermochemically responsive polymer films in the biomedical field is a promising direction, particularly for controllable drug delivery. However, the mechanism governing the shape memory effect (SME) remains poorly understood due to the complex interactions between solvent molecules and polymer chains. Herein, we integrate the Flory–Huggins theory with the transition state model to investigate the plasticization effect of solvent on the thermodynamic behavior of polymers. By introducing the concept of phase transition, the dependences of the glass transition temperature, thermomechanical properties, and shape memory behavior of polymer films on the thermochemical stimuli are discussed. Our theoretical analysis reveals that the entropy of mixing promotes the occurrence of the SME, while the enthalpy of mixing exerts an inhibitory effect on this process. Consequently, the competition between the entropy of mixing and the enthalpy of mixing is the critical factor determining whether polymers exhibit the SME under different thermochemical conditions. The effectiveness of the proposed model is further validated by applying it to predict the shape memory behavior of acrylate copolymer films under different thermochemical conditions. This study is expected to provide a practical methodology for understanding the working mechanism of the thermochemically driven SME in polymer films.

## 1. Introduction

Shape memory polymers (SMPs) are promising smart materials that can store large deformations and recover their original shapes in response to specific stimuli, such as temperature, pressure, light, and electromagnetic fields [1,2,3,4,5,6,7,8]. In recent decades, thermochemically driven SMPs have attracted considerable attention in biomedical applications due to their tunable thermomechanical properties, low cost, and biocompatibility [9,10,11]. For thermochemically driven SMPs, the temporary shape is stabilized through the vitrification of macromolecular chains during rapid cooling, while the stored strain is gradually released as the temperature approaches the glass transition temperature (*T_g_*) [12].

Specifically, the absorbed solvent molecules lower the *T_g_* of SMPs via chemical plasticization [13] or physical swelling [14], thereby enabling the SME with only a minor temperature increase or even without any temperature change. The physical swelling effect of the solvent is more widespread in promoting the SME in amorphous polymer films, as it allows for a broader range of material selections [15]. Lu et al. demonstrated that polystyrene SMPs exhibit solvent-induced shape recovery behavior through a swelling-induced reduction in *T_g_* [16]. Du et al. revealed that water enables faster shape recovery in poly(vinyl alcohol) compared to organic solvents due to stronger polymer–solvent mixing interactions [17]. Melocchi et al. confirmed the feasibility of a 3D-printed intravesical drug delivery system based on the swelling-induced shape memory mechanism [18]. Chemical plasticization represents another approach to inducing the SME in amorphous polymers. Huang et al. demonstrated that hydrogen bonding between water molecules and polyurethane chains can promote the SME [19]. Subsequently, Lu et al. investigated the glass transition behavior of polyurethane SMPs under the influence of water-induced chemical plasticization [20]. Their results demonstrate that physical swelling and chemical plasticization occur simultaneously in water–polyurethane mixtures. Furthermore, Ni et al. developed a four-dimensional printable shape memory hydrogel that operates through phase separation, providing a distinct mechanism for precise shape-shifting control [21]. This mechanism has been well investigated in our previous work [22].

However, the glass transition is fundamentally a kinetic relaxation phenomenon in SMPs. Furthermore, the thermodynamic interactions between solvent molecules and polymer segments are highly complex, which poses significant challenges in elucidating the working mechanism behind the thermochemically driven shape memory effect (SME). Therefore, it is crucial to develop a robust theoretical framework for thermochemically driven SMPs to guide their practical applications in the biomedical field [23,24,25].

Currently, constitutive models for SMPs are primarily classified into two types: physically based phase transition models [26,27,28,29,30] and phenomenological thermoviscoelastic models [31,32,33,34,35]. Based on the concept of phase transition, Lu et al. constructed a state diagram for solvent-driven SMPs at room temperature [36]. Subsequently, Li et al. introduced a temperature-dependent model incorporating the phase evolution law for SMPs [37,38]. Liu et al. developed a phase transition framework to characterize the SME of SMPs under finite deformation [39]. Based on the thermoviscoelastic approach, Qi et al. extended the standard linear solid model to describe the relaxation behavior of SMPs at different heating rates [40]. Xiao et al. further proposed a computational model to predict the shape fixity ratio of solvent-driven SMPs and implemented this model for finite element analysis [41]. Unfortunately, due to the complexity of component composition and driving environment, existing models struggle to fully understand the shape memory mechanism of amorphous polymers under thermochemically cooperative activation.

According to the phase transition model [27], the release of the temporary shape in amorphous polymers is primarily governed by their thermodynamic phase transition process, wherein the frozen phase gradually transforms into the active phase under external stimuli. To characterize the thermodynamic behavior of SMPs in complex thermochemical fields, we extend the Flory–Huggins theory [42] to quantitatively analyze the influences of temperature and solvent molecules on both the enthalpy and entropy of polymer chains. Subsequently, the free energy function is incorporated into the transition state theory [43] to systematically investigate the competitive effect of the enthalpy of mixing and the entropy of mixing on the shape memory behavior and thermomechanical properties of SMPs under different temperatures and solvent volume fractions. Furthermore, the accuracy of this model has been validated through existing experimental results [44]. It has been demonstrated that the ability of amorphous polymers to achieve the solvent-driven SME at different temperatures is predominantly influenced by the interaction parameter between solvent molecules and polymer chains.

There are three key findings in this work. (1) The solvent-driven SME in amorphous polymer films is primarily governed by the interaction parameter between solvent molecules and polymer chains. (2) The entropy of mixing facilitates the SME, while the enthalpy of mixing inhibits it, making their competition the critical determinant of SME occurrence under different thermochemical conditions. (3) This entropy–enthalpy balance can be fine-tuned by modifying the interaction parameter. Therefore, this physics-based model is expected to facilitate the understanding of the thermodynamic mechanism governing the thermochemically responsive behavior of SMPs, thereby providing valuable insights for the development of SMPs with the customizable SME.

## 2. Thermodynamic Model for SMPs Under Thermochemical Stimuli

Based on the phase transition model [27], the SME observed in amorphous polymers under external stimuli can be attributed to the microscopic phase evolution from the frozen state to the active state. As temperature increases, the frozen phase gradually transforms into the active phase, leading to the macroscopic recovery of the temporary shape. According to the transition state theory [45], the temperature-dependent transition rate (*k*) of SMPs can be expressed as follows:

(1) $$k(T)=k_{0}\exp\left(-\frac{\Delta G^{‡}}{RT}\right),$$

where *k*_0_ is a pre-exponential parameter proportional to the vibrational frequency of the polymer segments, *R* is the gas constant, *T* is the absolute temperature, and $\Delta G^{‡}$ is the Gibbs free energy of activation. 

The Gibbs free energy of SMPs comprises both the entropy of activation ($\Delta S^{‡}$) and the enthalpy of activation ($\Delta H^{‡}$) and can be expressed in the following form:

(2) $$\Delta G^{‡}=\Delta H^{‡}-T\Delta S^{‡}.$$

By substituting Equation (2) into Equation (1), we can determine that the transition rate of SMPs is governed by two thermodynamic parameters, namely $\Delta S^{‡}$ and $\Delta H^{‡}$:

(3) $$k(T)=k_{0}\exp\left(\frac{\Delta S^{‡}}{R}\right)\exp\left(-\frac{\Delta H^{‡}}{RT}\right).$$

Equation (3) only considers the effect of temperature on the phase transition rate. However, when SMPs are immersed in a solvent, the interaction between solvent molecules and polymer chains significantly influences both the enthalpy and entropy of the polymer system. Therefore, the transition rate of thermochemically responsive SMPs should be expressed as a function of both temperature and solvent volume fraction as follows:

(4) $$k(T,\phi_{s})=k_{0}\exp\left(\frac{\Delta S^{‡}+\Delta S_{m}(\phi_{s})}{R}\right)\exp\left(-\frac{\Delta H^{‡}+\Delta H_{m}(T,\phi_{s})}{RT}\right),$$

where $\Delta S_{m}$ and $\Delta H_{m}$ are the entropy and enthalpy of mixing, respectively.

**Figure 1:**
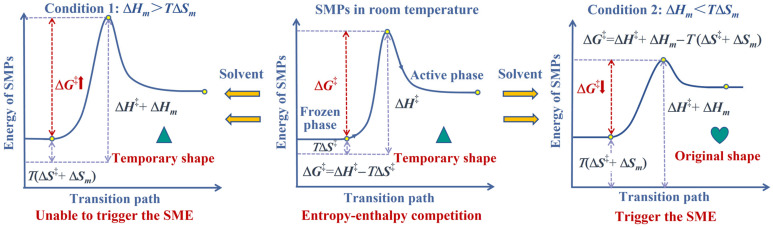
Schematic diagram of the thermodynamic mechanism behind the SME in thermochemically responsive SMPs.

According to the Flory–Huggins theory, the absorbed solvent molecules both increase the entropy of mixing and the enthalpy of mixing. As shown in Equation (4), the increase in the entropy of mixing promotes shape recovery in thermochemically responsive SMPs by accelerating their transition from the frozen phase to the active phase. Conversely, the change in the enthalpy of mixing hinders shape recovery by decelerating this phase transition. Therefore, there exists a competitive relationship between the effects of mixing entropy and mixing enthalpy on inducing the SME in amorphous polymers. As illustrated in Figure 1, the absorption of solvent molecules into the polymer chains leads to a simultaneous increase in both the entropy and enthalpy of mixing in SMPs. Therefore, it can be observed that the enthalpy of mixing and the entropy of mixing exhibit a competitive relationship in determining the shape memory behavior of amorphous polymers. When the contribution of the entropy of mixing exceeds that of the enthalpy of mixing, solvents are more likely to promote the SME at lower temperatures.

According to the Flory–Huggins theory [42], the relationship between the mixing of entropy ($\Delta S_{m}$) and the volume fraction of solvent in SMPs ($\phi_{s}$) can be written as

(5) $$\Delta S_{m}(\phi_{s})=-R\left(n_{s}\ln\phi_{s}+n_{m}\ln(1-\phi_{s})\right),$$

where $n_{s}$ and $n_{m}$ are the mole numbers of the solvent molecules and polymer chains in SMPs, respectively.

Based on the quasi-lattice model [46], the volume fraction of solvent in SMPs can be further expressed as follows:

(6) $$\phi_{s}=\frac{n_{s}}{n_{s}+xn_{m}},$$

where *x* is the volume ratio of the polymer chain relative to the solvent molecule.

By substituting Equation (6) into Equation (5), we can derive the relationship between the entropy of mixing and the volume fraction of solvent molecules as follows:

(7) $$\Delta S_{m}(\phi_{s})=-Rn_{m}\left(\frac{x\phi_{s}}{1-\phi_{s}}\ln\phi_{s}+\ln\left(1-\phi_{s}\right)\right).$$

Dayantis proposed the following relationship to characterize the dependence of the enthalpy of mixing on both temperature and the volume fraction of absorbed solvent [42]:

(8) $$\Delta H_{m}(T,\phi_{s})=R\;T\;\chi \;n_{s}(1-\phi_{s}),$$

where χ is the interaction parameter between solvent molecules and polymer segments. For thin polymer films, it is assumed that the diffusion of solvent molecules within the thin film follows a homogeneous diffusion process. Additionally, to more accurately describe the nonlinear interaction between polymers and solvent molecules, the interaction parameter is expressed as a function of both the volume fraction of solvent in SMPs and temperature as follows:

(9) $$\chi (T,\phi_{s})=\frac{A}{T}+B\phi_{s}+C,$$

where *A*, *B*, and *C* are the fitting parameters.

By substituting Equation (6) into Equation (8), we can further replace the variable $n_{s}$ with the constant $n_{m}$:

(10) $$\Delta H_{m}(T,\phi_{s})=\;R\;x\;Tn_{m}\;\chi \phi_{s}.$$

By substituting Equations (7) and (10) into Equation (4), we can obtain the final expression for the transition rate of SMPs under thermochemical stimuli:

(11) $$k(T,\phi_{s})=k_{0}\exp\left(\frac{\Delta S^{‡}}{R}-n_{m}\left(\frac{x\phi_{s}}{1-\phi_{s}}\ln\phi_{s}+\ln\left(1-\phi_{s}\right)\right)\right)\exp\left(-\frac{\Delta H^{‡}}{RT}-xn_{m}\chi \phi_{s}\right).$$

According to the transition state theory [43], the relaxation time (τ) of amorphous polymers is inversely proportional to the phase transition rate. Based on Equation (11), the relaxation time of SMPs under different thermochemical conditions can be determined as follows:

(12) $$\tau (T,\phi_{s})=\tau_{0}\underset{\mathrm{Entropy}\;\mathrm{effect}}{\underset{︸}{\exp\left(-\frac{\Delta S^{‡}}{R}+n_{m}\left(\frac{x\phi_{s}}{1-\phi_{s}}\ln\phi_{s}+\ln\left(1-\phi_{s}\right)\right)\right)}}\underset{\mathrm{Enthalpy}\;\mathrm{effect}}{\underset{︸}{\exp\left(\frac{\Delta H^{‡}}{RT}+xn_{m}\chi \phi_{s}\right),}}$$

where $\tau_{0}=1/k_{0}$.

The relaxation time of amorphous polymers can also be characterized using the apparent activation energy ($\Delta H_{a}$) in the following form:

(13) $$\tau (T,\phi_{s})=\tau_{0}\exp\left(-\frac{\Delta H_{a}(T,\phi_{s})}{RT}\right).$$

By comparing Equations (12) and (13), the expression for the apparent activation energy can be derived as follows:

(14) $$\Delta H_{a}(T,\phi_{s})=\Delta G^{‡}+n_{m}RT\left(\frac{x\phi_{s}}{1-\phi_{s}}\ln\phi_{s}+\ln\left(1-\phi_{s}\right)+x\chi \phi_{s}\right).$$

It is important to highlight that the classical transition state theory cannot directly describe the segmental dynamics of amorphous polymers, as polymer segments are constrained by their surrounding motion units near the glass transition temperature. Consequently, Adam et al. proposed that these motion units exhibit cooperative relaxation during the glass transition process [47]. This is evidenced by the observation that the activation energy decreases with increasing temperature [48]. Therefore, in this study, the transition state theory is extended by expressing the activation energy as a function of temperature and the volume fraction of absorbed solvent, thereby enabling a more comprehensive description of the glass transition behavior of amorphous polymers under thermochemical stimuli. Based on the phase transition model [27], the shape recovery rate of SMPs is equal to their phase transition rate. Therefore, the volume fraction of the frozen phase ($\varphi_{f}$) in SMPs can be written as

(15) $$\varphi_{f}(T,\phi_{s})=\frac{\varepsilon_{s}}{\varepsilon_{pre}}={\left(1-\exp\left(-\frac{\Delta H_{a}(T,\phi_{s})}{RT}\right)\right)}^{\frac{\Delta t}{\tau_{0}}},$$

where Δt is the heating time, and *τ*_0_ is the internal time scale.

By substituting Equation (14) into Equation (15), the release of the stored strain ($\varepsilon_{s}$) can be expressed as a function of temperature and the volume fraction of absorbed solvent in the following manner:

(16) $$\begin{array}{l} \varepsilon_{s}(T,\phi_{s})=\varepsilon_{pre}{\left(1-\exp\left(-\frac{\Delta G^{‡}}{RT}-n_{m}\left(\frac{x\phi_{s}}{1-\phi_{s}}\ln\phi_{s}+\ln\left(1-\phi_{s}\right)\right)-xn_{m}\chi \phi_{s}\right)\right)}^{\frac{\Delta t}{\tau_{0}}}. \end{array}$$

**Figure 2:**
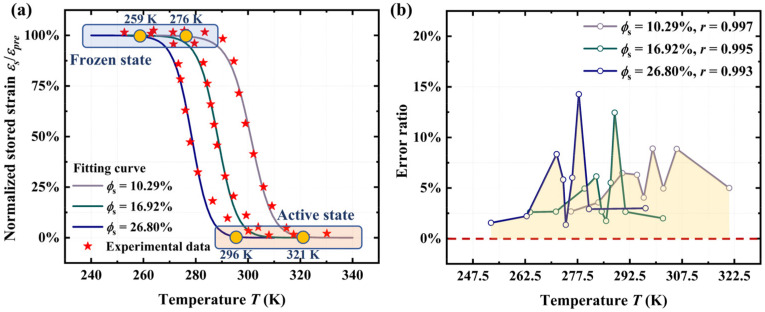
(**a**) Comparison of the numerical results from Equation (16) with experimental data [44] on the stored strain of acrylate copolymer films at different volume fractions of IPA. (**b**) Error ratio and correlation coefficient from the comparison between numerical results and experimental observations.

As shown in Figure 2, the numerical results from Equation (16) are compared with the experimental data on the thermochemically induced shape recovery of acrylate copolymer films [44]. According to Ref. [44], acrylate copolymer films were synthesized by mixing tert-butyl acrylate, poly(ethylene glycol) dimethacrylate, and photo-initiator 2, 2-dimethoxy-2-phenylacetophenone at a weight ratio of 98%:2%:0.2%, followed by curing in a UV oven for 20 min. Isopropyl alcohol (IPA) was used as the driving solvent. To achieve acrylate copolymers with different concentrations of IPA, film specimens were immersed in IPA for different durations (10 min, 30 min, and 1 h). Based on the swelling results of the acrylate copolymer in IPA solvent [44], the corresponding volume fractions of IPA in polymer systems were determined to be 10.29%, 16.92%, and 26.80%, respectively. Subsequently, the experimental data were extracted using the image digitization tool in OriginPro software (version number: 9.9.0.225). Dynamic mechanical analysis (DMA) revealed that the modulus of the glassy state of the acrylate copolymers were 870.03 MPa, 1250.22 MPa, and 1375.32 MPa, corresponding to IPA volume fractions of 26.80%, 16.92%, and 10.29%, respectively [44]. In addition, free shape recovery tests of the acrylate copolymers indicated that heating times were 1110 s, 1170 s, and 1350 s, corresponding to IPA volume fractions of 26.80%, 16.92%, and 10.29%, respectively.

During the calculations, the experimental data on the free shape recovery behavior of the acrylate copolymer at an IPA volume fraction of 10.29% were first employed to determine the parameter values. The Universal Global Optimization (UGO) method implemented in 1Stopt software (version number: 9.0) was applied to determine the parameter values used in Equation (16). The convergence judgment index was set to 1 × 10^−10^, and the maximum number of iterations was limited to 1000. In addition, although the interaction parameter (*χ*) is a function of temperature and the concentration of the IPA solvent in SMPs, its reasonable range typically lies between 0.5 and 1. When the value of the interaction parameter falls below 0.5, the interaction between the polymer and the solvent becomes relatively strong, potentially causing the polymer to completely dissolve in the solvent. If the value of the interaction parameter exceeds 1, the polymer chains tend to contract, which inhibits the swelling process of SMPs. Moreover, the volume ratio of polymer segments to solvent molecules should significantly exceed 1. To validate the accuracy of the proposed model, we characterize the evolution of the glass transition temperature and the storage modulus for the acrylate copolymer upon thermochemically coupling activation, using the same parameter values listed in Table 1.

Figure 2b shows that the numerical results from the proposed model are in excellent agreement with the experimental observations [44]. The correlation coefficients (*r*) between the fitting results from Equation (16) and the experimental data for acrylate copolymer films at IPA volume fractions of 10.29%, 16.92%, and 26.80% are 0.997, 0.995, and 0.993, respectively. The error ratio is calculated as the ratio of the absolute difference between the experimental value and the theoretical value to the experimental value.

It is crucial to highlight that the constant pressure condition was applied when solving for variations in Gibbs free energy. Under this condition, the enthalpy change of the polymer system corresponds precisely to the energy absorbed or released by the system; otherwise, the equation becomes unsolvable under thermochemical coupling stimuli. Therefore, this theory primarily focuses on the free shape recovery behavior of polymer thin films, while the effects of polymer geometries and external constraints on the Gibbs free energy of SMPs were neglected in the calculation process. In practical applications, these materials may encounter external constraints, thereby leading to potential discrepancies between theoretical predictions and real-world behavior. In addition, the proposed model is primarily employed to characterize the solvent-driven shape memory behavior of polymer thin films. Therefore, it is assumed that the solvent is uniformly distributed within the polymer systems. In practical applications, this model may not be suitable for characterizing the SME in bulk materials due to heterogeneous swelling [49].

As illustrated in Figure 2a, the initial shape memory temperature of the acrylate copolymer decreases from 276 K to 254 K as the volume fraction of absorbed IPA increases from 10.29% to 26.80%. According to the proposed model, this trend can be attributed to the reduction in the Gibbs free energy required for the activation of SMPs with an increase in the volume fraction of the absorbed solvent. Based on the proposed model, we can precisely control the shape memory temperature range of thermochemically induced SMPs by regulating the volume fraction of the absorbed solvent, thereby fulfilling specific engineering requirements. However, the proposed model is not suitable for characterizing the shape memory behavior of gel systems. When the volume fraction of absorbed solvent is excessive, the influence of solvent molecules on the shape fixation rate will increase significantly.

**Figure 3:**
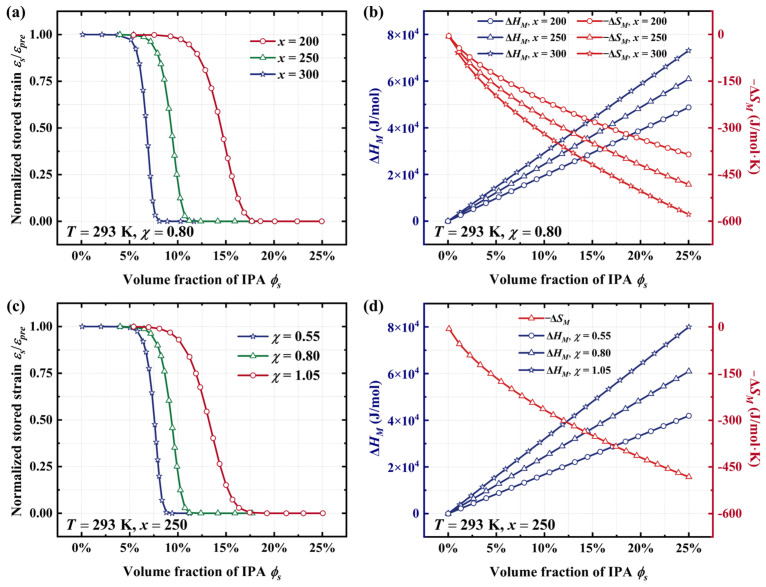
(**a**) Numerical results and (**b**) thermodynamic mechanisms of the normalized strain recovery for acrylate copolymer films at different volume ratios of polymer segments to solvent molecules. (**c**) Numerical results and (**d**) thermodynamic mechanisms of the normalized strain recovery for acrylate copolymer films at different values of the interaction parameter.

The effects of thermodynamic parameters on the free recovery behavior of SMPs are further investigated using the proposed model. Figure 3a illustrates that as the volume ratio of polymer chains to solvent molecules (*x*) increases, a greater number of solvent molecules are required to be absorbed into the SMPs to activate the SME. According to the proposed model, we further investigate the thermodynamic mechanism underlying this phenomenon. As shown in Figure 3b, with the increase in the value of parameter x, the enthalpy of mixing for the polymer–solvent system exhibits a significant rise when absorbing the same number of IPA molecules, while the entropy of mixing demonstrates a notable decrease. Based on Equation (11), when the enthalpy of mixing is the dominant factor, the probability of SMPs transforming from the frozen phase into the activated one is significantly reduced. Consequently, it becomes challenging to achieve the SME in thermochemically responsive SMPs at high values of the volume ratio of polymer chains to solvent molecules.

On the other hand, Figure 3c investigates the interactive effect between solvent molecules and polymer segments on the shape memory behavior of amorphous polymers. Numerical results indicate that a low value of the interaction parameter promotes the thermochemically induced SME. The physical mechanism behind this phenomenon is investigated using the proposed model. As shown in Figure 3d, the enthalpy of mixing is significantly influenced by the interaction parameter (χ) between solvent molecules and polymer chains. Specifically, the enthalpy of mixing increases from 4.2 × 10^4^ J/mol to 8.0 × 10^4^ J/mol as the interaction parameter rises from 0.55 to 1.05. However, numerical results indicate that the value of the entropy of mixing remains constant across different values of the interaction parameter. According to the transition state theory [43], an increase in the enthalpy of mixing is unfavorable for the phase transition in solvent–polymer mixtures, thereby requiring a greater absorption of solvent molecules to achieve the SME at the same temperature.

## 3. Thermomechanical Behavior and Glass Transition Temperature

During the phase evolution, the storage modulus is a crucial parameter for characterizing the shape memory behavior of SMPs. Therefore, it is essential to investigate the thermomechanical properties of SMPs under various thermochemical stimuli to advance their applications in the biomedical field. To address this issue, we integrate the phase transition model [27] with the Mori–Tanaka framework [28] to describe the relationship between the storage modulus, temperature, and solvent volume fraction in SMPs as follows:

(17) $$E(T,\phi_{s})=E_{f}\left(1+\frac{\varphi_{a}(T,\phi_{s})\left(E_{a}/E_{f}-1\right)}{1+\omega \varphi_{f}(T,\phi_{s})\left(E_{a}/E_{f}-1\right)}\right),$$

where *w* is a material constant associated with Poisson’s ratio.

**Figure 4:**
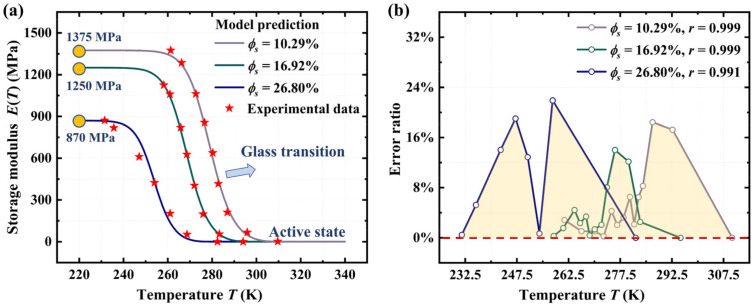
(**a**) Comparison of the numerical results from Equation (17) with experimental results [44] for the thermomechanical behavior of acrylate copolymer films at different volume fractions of absorbed IPA solvent. (**b**) Error ratio and correlation coefficient from the comparison between numerical results and experimental observations.

Based on the parameter values listed in Table 1, we further employ Equation (17) to investigate the influence of absorbed IPA on the thermomechanical behavior of the acrylate copolymer film. The fitting value of the parameter *w* is 0.97. Figure 4a shows the predictions are in good agreement with the experimental results [44] of the storage modulus of the acrylate copolymer at IPA volume fractions of 10.29%, 16.92%, and 26.80%, respectively. Figure 4b shows a correlation coefficient (*r*) exceeding 0.991, thereby validating the accuracy and reliability of the proposed model. Furthermore, Figure 4a shows that the storage modulus of SMPs exhibits a significant decrease upon the glass transition from the frozen state to the active state. Meanwhile, numerical results indicate that the chemical plasticizing effect of solvent molecules significantly reduces the storage modulus of SMPs at the same temperature. This thermochemically cooperative activation promotes the SME of amorphous polymers at relatively low temperatures, thereby enhancing their suitability for biomedical applications. Since amorphous polymers exhibit the SME upon glass transition, the proposed model is specifically designed for investigating the thermomechanical properties of SMPs within the glass transition temperature range. The temperature-dependent thermomechanical behavior of amorphous polymers in the glassy state has been discussed in Ref. [50].

**Figure 5:**
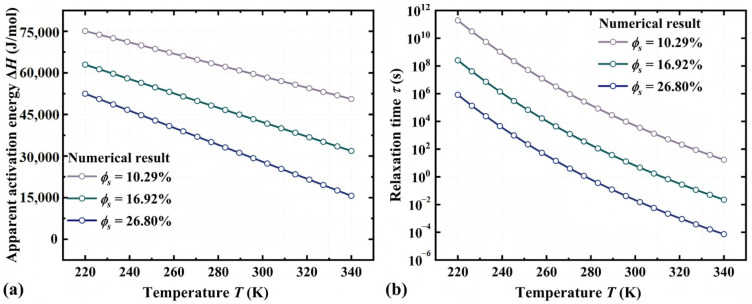
Numerical results for (**a**) the apparent activation energy and (**b**) the relaxation time of acrylate copolymer films under different thermochemical stimuli.

Based on the proposed model, we further explore the working mechanism behind the glass transition in thermochemically responsive SMPs. As shown in Figure 5a, the apparent activation energy is the key microscopic factor governing the glass transition of SMPs. Specifically, as the temperature increases from 220 K to 340 K, the apparent activation energy of the acrylate copolymer film decreases from 7.51 × 10^4^ J/mol to 5.06 × 10^4^ J/mol when the volume fraction of IPA solvent is 10.29%. Furthermore, the apparent activation energy significantly decreases from 5.06 × 10^4^ J/mol to 1.57 × 10^4^ J/mol with an increase in the volume fraction of IPA solvent from 10.29% to 26.80% at the temperature of 340 K. Therefore, it can be concluded that the glass transition and the associated release of the stored strain are driven by the decrease in the apparent activation energy ($\Delta H_{a}$), which can be well characterized using the proposed model.

The macroscopic manifestation of the glass transition in SMPs is characterized by a significant reduction in relaxation time. Based on Equation (12), Figure 5b illustrates the relationship between the relaxation time of SMPs, temperature, and the volume fraction of the IPA solvent. It is found that the relaxation time decreases markedly with an increase in the temperature and the volume fraction of the IPA solvent. According to Ref. [34], the SME in amorphous polymers arises from the entropy–driven relaxation behavior of their polymer segments. Therefore, it can be concluded that the shape memory behavior of thermochemically responsive polymers is fundamentally induced by the fact that both thermal and chemical stimuli can reduce the relaxation time. This enables the observation of the SME within the experimental time scale.

The glass transition temperature (*T_g_*) is another thermodynamic parameter for characterizing the shape memory behavior of SMPs. When the temperature is below *T_g_*, the temporary shape is fixed due to the vitrification of the polymer segments. As the temperature approaches *T_g_*, the temporary shape can recover along with the relaxation behavior of SMPs. From a thermodynamic perspective, the relaxation time of SMPs at *T_g_* is approximately 100 s. Using Equation (12), we can build the relationship between *T_g_*, temperature, and the volume fraction of absorbed solvent as follows…

(18) $$T_{g}(T,\phi_{s})=\frac{\Delta G^{‡}}{R}{\left(\ln\left(\frac{100}{\tau_{0}}\right)-n_{m}\left(\frac{x\phi_{s}}{1-\phi_{s}}\ln\phi_{s}+\ln\left(1-\phi_{s}\right)+x\chi \phi_{s}\right)\right)}^{-1},$$

**Figure 6:**
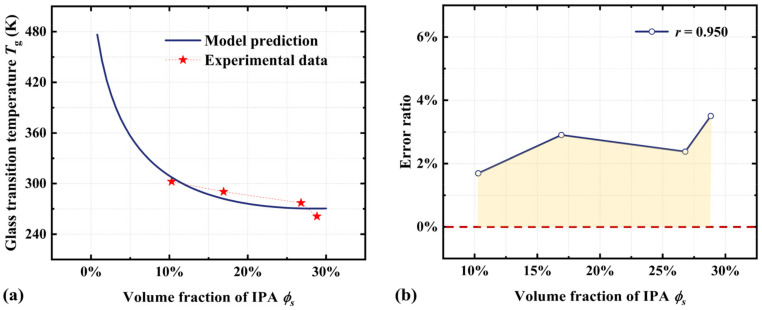
(**a**) Comparisons of the numerical results from Equation (18) with experimental results [44] for the *T_g_* of the acrylate copolymer film as a function of the volume fraction of absorbed IPA solvent. (**b**) Error ratio and correlation coefficient from the comparison between numerical results and experimental observations.

Based on the values of parameters listed in Table 1, we further predict the *T_g_* of the acrylate copolymer film as a function of the volume fraction of IPA solvent. As shown in Figure 6, *T_g_* decreases gradually from 482 K to 241 K as the volume fraction of absorbed IPA solvent increases from 2.5% to 40%. Numerical results also indicate that the acrylate copolymer film can exhibit the SME at room temperature when the volume fraction of absorbed IPA solvent exceeds 15%. This model provides an effective approach for driving the SME at different temperatures by simply changing the volume fraction of the absorbed solvent in SMPs.

**Figure 7:**
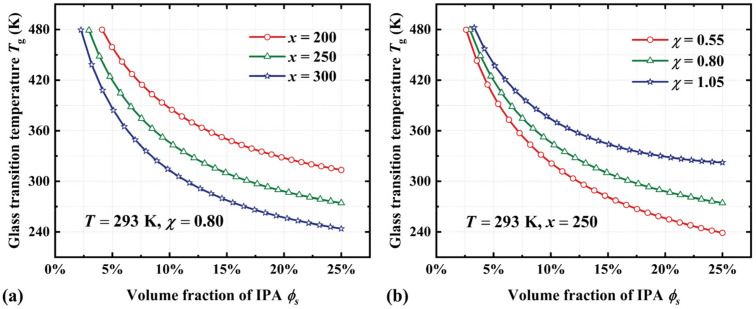
Numerical results for the *T_g_* of the acrylate copolymer film as a function of the volume fraction of IPA. (**a**) *x* = 200, 250, and 300. (**b**) *χ* = 0.55, 0.80, and 1.05.

Based on Equation (18), we investigate the significant impact of thermodynamic parameters (*x* and *χ*) on the *T_g_* of thermochemically responsive SMPs. Figure 7a shows the *T_g_* of the acrylate copolymer film at the value of *x* of 200, 250, and 300, respectively. The numerical results indicate that the *T_g_* increases markedly with a decrease in the volume ratio of polymer segments to absorbed IPA solvent molecules. Additionally, the interaction parameter between solvent molecules and polymer segments (*χ*) exerts a substantial influence on the *T_g_* of SMPs. Specifically, the *T_g_* decreases from 322 K to 239 K as *χ* decreases from 1.05 to 0.55. Therefore, it can be concluded that the thermochemically induced SME in amorphous polymers is primarily governed by these above thermodynamic parameters. This study provides a theoretical foundation for the further development and practical applications of thermochemically responsive SMPs.

In the preceding analysis, the classical Flory–Huggins theory is employed to describe the homogeneous mixing of solvent molecules and polymer chains. However, in specific cases, such as the water-driven shape memory process in polyurethane [19], both the physical swelling effect and the chemical plasticizing effect operate synergistically, leading to a reduction in the *T_g_* of SMPs. Based on this foundation, we further categorize the absorbed solvent into two types, bound solvent and free solvent:

(19) $$\phi_{s}=\phi_{s,b}+\phi_{s,f},$$

where $\phi_{s,b}$ and $\phi_{s,f}$ are the volume fraction of bound solvent and free solvent, respectively. The free solvent is absorbed into polymer systems owing to the physical swelling effect. Following Equation (18), the reduction in *T_g_* induced from the free solvent can be written as,

(20) $$T_{g0}(\phi_{s,f})=\frac{\Delta G^{‡}}{R}{\left(\ln\left(\frac{100}{\tau_{0}}\right)-n_{m}\left(\frac{x\phi_{s,f}}{1-\phi_{s,f}}\ln\phi_{s,f}+\ln\left(1-\phi_{s,f}\right)+x\chi \phi_{s,f}\right)\right)}^{-1}.$$

On the other hand, the bound solvent is absorbed into SMPs via chemical plasticization. According to Ref. [51], the additional influence of the bound solvent on the *T_g_* can be described as follows:

(21) $$T_{g}(\phi_{s})=T_{g0}(\phi_{s,f})\left(1-\frac{\phi_{s,b}}{1+\exp(\Delta E_{b}/RT)}\right),$$

where $\Delta E_{b}$ is the activation energy required to form the hydrogen bonding.

Based on the proposed model, we further analyze both the physical swelling effect and the chemical plasticization effect of water on the *T_g_* of polyurethane SMP. According to Ref. [19], hydrogen bonding is identified as the key parameter promoting water absorption in polyurethane SMP, and the absorbed water is categorized into two types: bound water and free water. The bound water forms through hydrogen bonding, while the free water infiltrates into the polymer system via physical swelling. The content of bound water is determined by heating the water–polyurethane mixtures to 120 °C [19]. The volume fraction of water in the polymer system that remains non-evaporated is considered as the bound water.

**Figure 8:**
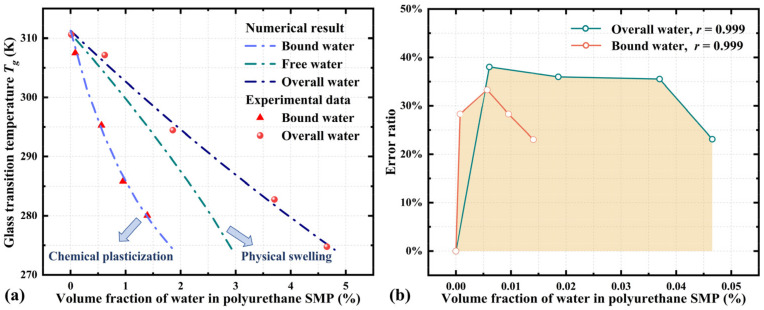
(**a**) Comparison of the numerical results from Equation (21) with experimental results [19] for the glass transition behavior of polyurethane SMP. (**b**) Error ratio and correlation coefficient from the comparison between the numerical results and experimental observations.

As depicted in Figure 8a, Equation (21) characterizes well these dual effects on the glass transition behavior of polyurethane SMP. The parameter values used in Equation (21) are listed in Table 2. The interaction parameter is set as a constant since the temperature remains at room level and the variation in the volume fraction of water in polyurethane is minimal. It is evident that the infiltration of free water into the polyurethane SMP is sustained due to the chemical plasticization induced by the bound water. Consequently, the chemical plasticization effect reduces *T_g_*, enabling the efficient release of stored mechanical energy at relatively low temperatures. Furthermore, Figure 8b demonstrates a correlation coefficient exceeding 0.999 between the numerical results and experimental data [19], validating the applicability of the proposed model for describing both the physical swelling and chemical plasticization effects of solvents on the glass transition of SMPs.

## 4. Effect of Kinetic Diffusion of Solvent Molecules on the SME

The above analysis focuses on the thermodynamic behavior of SMPs. However, understanding the kinetics of SME activation is crucial for elucidating the shape recovery dynamics in amorphous polymers. To solve this issue, Fick’s second law is employed to characterize the time-dependent solvent diffusion in polymer systems. The relative volume stretch of SMPs (*w*(*t*)) immersed in solvent can be expressed as follows:

(22) $$w(t)=\frac{v\left(t\right)-v_{0}}{v_{\infty }-v_{0}},$$

where *v*(*t*) is the volume of SMPs at time *t*, *v*_0_ is the initial volume, and $v_{\infty }$ is the volume at time $t_{\infty }$.

Based on the Crank form of Fick’s second law for thin films [52], the relative volume stretch can be written as

(23) $$w(t)=1-\sum_{n=0}^{\infty }\frac{8}{{\left(2n+1\right)}^{2}\pi^{2}}\exp\left(-D\frac{{\left(2n+1\right)}^{2}\pi^{2}t}{h}\right),$$

where *h* is the diffusion length, and *D* is the diffusion coefficient.

Equation (23) cannot be solved numerically. According to Ref. [52], the relative volume stretch can be further simplified under the condition of short observation times as follows:

(24) $$w(t)=\frac{2}{\sqrt{\pi }}\frac{\sqrt{D}}{h}\sqrt{t},$$

As revealed in Ref. [53], the diffusion coefficient is a function of both temperature and the volume fraction of solvent in SMPs:

(25) $$D(T,\phi_{s})=D_{0}\left(T\right)\left(1+k_{d1}\phi_{s}+k_{d2}\phi_{s}^{2}\right),$$

where $k_{d1}$ and $k_{d2}$ are fitting parameters. The relationship between the diffusion coefficient and temperature can be quantitatively described using the Eyring expression:

(26) $$D_{0}(T)=D^{*}\exp\left(-\frac{E_{d}}{RT}\right),$$

where D* is a constant, and *E_d_* is the activation energy of diffusion.

Combining Equations (24) and (25), the diffusion coefficient of SMPs can be written as

(27) $$D(T,\phi_{s})=D^{*}\exp\left(-\frac{E_{d}}{RT}\right)\left(1+k_{d1}\phi_{s}+k_{d2}\phi_{s}^{2}\right).$$

Based on Equation (22), the relationship between the relative volume stretch (*w*(*t*)) and the volume fraction of solvent molecules in SMPs ($\phi_{s}$) is expressed as follows:

(28) $$w(t)=\frac{v_{0}{\left(1-\phi_{s}\right)}^{-1}-v_{0}}{v_{\infty }-v_{0}}.$$

Combining Equations (24) and (28), the kinetics of solvent diffusion in SMPs can be described using the following expression:

(29) $$\phi_{s}(t)=1-{\left(\frac{v_{\infty }-v_{0}}{v_{0}}\frac{2}{\sqrt{\pi }}\frac{\sqrt{D}}{h}\sqrt{t}+1\right)}^{-1}.$$

Furthermore, the diffusion-limited effect on the volume of SMPs at time $t_{\infty }$ is modeled from the perspective of the Gibbs free energy. During the swelling process, the change in free energy can be decomposed into two components: the mixing free energy between the polymer and the solvent ($\Delta G_{m}$) and the elastic free energy of the polymer network ($\Delta G_{d}$). Combining Equations (5) and (8), the mixing free energy can be expressed as follows:

(30) $$\Delta G_{m}=RT(n_{s}\ln\phi_{s}+n_{m}\ln\phi_{m}+\chi n_{s}\phi_{m}).$$

According to the statistical theory of elasticity [54], the elastic free energy of SMPs has the following form:

(31) $$\Delta G_{d}=\frac{1}{2}Nk_{b}T\left(\lambda_{1}^{2}+\lambda_{2}^{2}+\lambda_{3}^{2}-3\right).$$

where *λ* is the ratio of the lengths of each side of SMPs after swelling relative to their original lengths, *k_b_* is Boltzmann’s constant, and *N* is the degree of crosslinking. For the isotropic polymer film, we can obtain the following expression:

(32) $$\lambda_{1}=\lambda_{2}=\sqrt{\frac{v(t)}{v_{0}}}=\sqrt{\frac{1}{1-\phi_{s}}},\;\lambda_{3}=1.$$

When the polymer reaches swelling equilibrium, the chemical potential of the solvent within the swollen polymer becomes equal to that of the external solvent, i.e., Δμ=0,

(33) $$\Delta \mu =\frac{\partial \Delta G_{m}}{\partial n_{s}}+\frac{\partial \Delta G_{d}}{\partial n_{s}}=\ln\phi_{s,\infty }+\left(1-\phi_{s,\infty }\right)+\chi {\left(1-\phi_{s,\infty }\right)}^{2}+\frac{Nk_{b}T}{xn_{m}}=0.$$

where $\phi_{s,\infty }$ is the volume fraction of solvent molecules in SMPs at time $t_{\infty }$. Therefore, the parameter $v_{\infty }$ can be written as

(34) $$v_{\infty }=\frac{v_{0}}{1-\phi_{s,\infty }}.$$

Substituting Equation (34) into Equation (33), the volume expression of SMPs under the diffusion-limited condition can be derived. This expression is further applied in the calculation of Equation (29):

(35) $$\ln\left(1-\frac{v_{0}}{v_{\infty }}\right)+\frac{v_{0}}{v_{\infty }}+\chi {\left(\frac{v_{0}}{v_{\infty }}\right)}^{2}+\frac{Nk_{b}T}{xn_{m}}=0.$$

**Figure 9:**
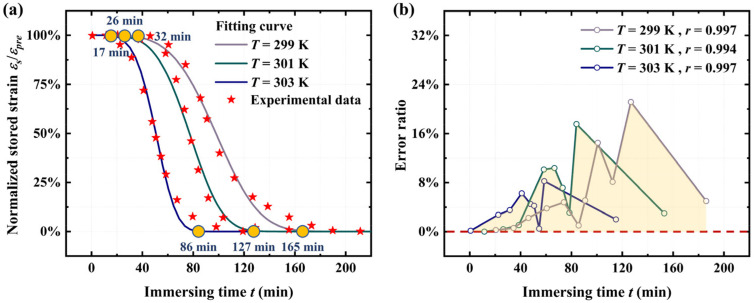
(**a**) Comparison of the numerical results from Equations (19) and (29) with experimental results [55] for the time-dependent shape recovery behavior of the polyurethane under moisture conditions. (**b**) Error ratio and correlation coefficient from the comparison between the numerical results and experimental observations.

Combining Equations (19) and (29), we further analyze the kinetics of SME activation in amorphous polymer films to elucidate the shape memory dynamics. The theoretical model is employed to predict the time-dependent shape recovery of the polyurethane under moisture conditions [55]. The moisture-responsive polyurethane was prepared from the monomers of 1,6-hexamethylene diisocyanate, 1,4-butanediol, and N,N-bis(2-hydroxylethyl) isonicotinamide. The thickness of the polyurethane film is 0.5 mm, and the initial volume of polyurethane is 100 mm^3^ [55]. Free shape recovery tests show that the recovery time is 7980.42 s at 299 K, 6061.59 s at 301 K, and 4142.76 s at 303 K [55]. Other parameter values are listed in Table 3. During the calculations, experimental data on the shape recovery of the polyurethane thin film at 303 K were first employed to determine the parameter values used in Equations (19) and (29). To validate the accuracy of the proposed model, the same parameter values were applied to predict the shape recovery process of the polyurethane thin film at *T* = 301 K and *T* = 303 K. As shown in Figure 9b, the numerical predictions exhibit excellent agreement with the experimental data, with an error ratio below 21.8% and a correlation coefficient exceeding 0.994. Comparisons indicate that the initial shape recovery time increases from 17 min to 32 min as the temperature decreases from 303 K to 299 K. Moreover, the final shape recovery time of the polyurethane thin film increases from 86 min to 165 min under the same temperature reduction. Based on the proposed model, this phenomenon can be attributed to the dominance of the entropy of mixing over the enthalpy of mixing at higher temperatures, thereby reducing the diffusion-limited effect. Consequently, the polyurethane exhibits a faster moisture-driven shape recovery process at higher temperatures, which is effectively captured by the proposed model.

## 5. Conclusions

In this study, a phase-transition model was developed to elucidate the thermodynamic mechanism underlying the thermochemically induced SME in amorphous polymer films from the perspective of the change in Gibbs free energy. The chemical plasticizing effect of solvent molecules on the glass transition temperature, shape memory behavior, and thermomechanical properties of SMPs was investigated by combining the transition state theory and the Flory–Huggins model. The proposed model effectively characterized the thermochemically cooperative activation behavior of SMPs. Numerical results indicate that the occurrence of the thermochemically induced SME is governed by the competition between the entropy of mixing and the enthalpy of mixing in SMPs. Furthermore, it was found that this entropy–enthalpy competition can be modulated by changing the interaction parameter between solvent molecules and polymer chains. By accounting for the potential chemical reactions between the solvent and polymer chains, the proposed model demonstrated the ability to characterize the glass transition behavior of SMPs that exhibit both physical swelling and chemical plasticization effects. However, it is worth noting that the current model struggles to characterize the shape memory behavior induced by the phase separation between solvent and polymer chains. To address this limitation, future research could incorporate the phase separation mechanism into the proposed enthalpy–entropy competition framework, thereby enabling a more comprehensive description of the complex shape memory processes of SMPs governed by phase separation.

## Figures and Tables

**Table 1:** Values of the parameters used in Equation (16).

Parameter	Physical Meaning	Value	
$n_{m}(\mathrm{mol})$	Mole number of polymer chains in SMPs	0.50	
$\Delta G^{‡}(J/\mathrm{mol})$	Gibbs free energy	1.20 × 10^5^	
A (K)	Characterizing the temperature dependence of the interaction parameter	12.05	
B	Characterizing the concentration dependence of the interaction parameter	0.13	
C	Fitting constant used in Equation (9)	0.75	
x	Volume ratio of polymer segments to solvent molecules	274.08	
Δt (s)	Heating time [44]	*ϕ_s_* = 10.29%	1350
		*ϕ_s_* = 16.92%	1170
		*ϕ_s_* = 26.80%	1110
$\tau_{0}$ (s)	Internal time scale	*ϕ_s_* = 10.29%	1.38 × 10^−7^
		*ϕ_s_* = 16.92%	1.10 × 10^−7^
		*ϕ_s_* = 26.80%	1.02 × 10^−6^
*E_f_* (MPa)	The modulus of the frozen phase [44]	*ϕ_s_* = 10.29%	1375.32
		*ϕ_s_* = 16.92%	1250.22
		*ϕ_s_* = 26.80%	870.03
*E_a_* (MPa)	The modulus of the active phase [44]	1.53	
ω	Material constant used in the Mori–Tanaka model	0.97	

**Table 2:** Values of the parameters used in Equation (21).

Parameter	Physical Meaning	Value
$\Delta G^{‡}\;(J/\mathrm{mol})$	Gibbs free energy	3784.36
$\tau_{0}\;(s)$	Internal time scale	6.10 × 10^−4^
*x*	Volume ratio of polymer segments to solvent molecules	50.03
χ	Interaction parameter	0.71
*n_m_* (mol)	Mole number of polymer chains in SMPs	0.36
$\Delta E_{b}\;(J/\mathrm{mol})$	Activation energy required to form the hydrogen bonding	7.31

**Table 3:** Values of the parameters used in Equations (19) and (29).

Parameter	Physical Meaning	Value	
$\Delta G^{‡}\;(J/\mathrm{mol})$	Gibbs free energy	1.42 × 10^5^	
$\tau_{0}\;(s)$	Internal time scale	6.77 × 10^−10^	
*x*	Volume ratio of polymer segments to solvent molecules	83.36	
χ	Interaction parameter	0.60	
*n_m_* (mol)	Mole number of polymer chains in SMPs	0.99	
$E_{d}\;(\mathrm{kJ}/\mathrm{mol})$	The activation energy of diffusion	11.99	
v_0_ (mm^3^)	The initial volume of SMPs [55]	100	
*h* (mm)	Diffusion length [55]	0.5	
D*	A constant used in Equation (27)	6.45 × 10^−11^ m^2^/s	
Δt (s)	Diffusion time [55]	*T* = 299 K	7980.42
		*T* = 301 K	6061.59
		*T* = 303 K	4142.76

## Data Availability

The original contributions presented in this study are included in the article.
